# Bilateral Occurrence of Mandibular Permanent Second Molar With Four Roots: A Case Report and Literature Review

**DOI:** 10.1155/crid/4757607

**Published:** 2026-02-18

**Authors:** Agnieszka Chamarczuk, Laurentia Schuster, Till Dammaschke, Mariusz Lipski

**Affiliations:** ^1^ Department of Preclinical Conservative Dentistry and Preclinical Endodontics, Pomeranian Medical University in Szczecin, Szczecin, Poland, pum.edu.pl; ^2^ Department of Periodontology and Operative Dentistry, University of Münster, Münster, Germany, uni-muenster.de

**Keywords:** anatomic variation, cone-beam computed tomography, mandible, molar, tooth abnormalities, tooth root

## Abstract

Basic knowledge of tooth anatomy is essential for endodontic success. The most common configuration of the mandibular second molar is the presence of two roots. However, single‐, three‐, four‐, and even five‐rooted forms have also been reported in the literature. The purpose of this study is to present a clinical case of a mandibular second molar with four roots. Morphology was assessed using CBCT examination, which revealed that the patient has bilateral four‐rooted second lower molars—an anomaly that, to our knowledge, is extremely rare in the literature. Awareness and careful diagnosis of such anomalies are crucial to ensure all canals are treated, improving clinical outcomes and preventing treatment failure.

## 1. Introduction

The success of endodontic treatment depends on thorough preparation of the root canal, removal of bacterial biofilm, and disinfection and filling of the entire root canal system with an appropriate material. To achieve this, the dentist must have detailed knowledge of root canal morphology and its variations [1, 2]. Mandibular second molars present considerable anatomical and morphological diversity in terms of the number of roots and root canals. Most commonly, they have two roots—a mesial and a distal root—but single‐rooted and three‐rooted forms are also observed [3, 4]. The distal root most often exhibits a Vertucci Type I canal system, whereas the mesial root shows greater morphological variation, usually Vertucci Type IV or II, with distinct mesiobuccal and mesiolingual canals. When a third canal is present in the mesial root, it lies centrally and is termed the middle mesial canal [5–7]. In three‐rooted second molars, the additional root may be located lingually (radix entomolaris) or buccally (radix paramolaris) [7]. In single‐rooted second molars, the canal system may take the form of a C‐shaped canal. This morphology is named after the shape of the canal orifices, which form a C at the chamber floor, with the convexity directed either buccally or lingually. A defining feature of the C‐shaped canal system is the interconnection of the canals by isthmuses, sometimes extending along the entire root length and sometimes confined to a particular segment [8, 9]. A rarer anatomical variation is the four‐rooted second molar, with roots described as mesiobuccal, mesiolingual, distobuccal, and distolingual [10, 11]. Even more unusual, reports also exist of a five‐rooted mandibular second molar [12].

The presence of additional roots has important clinical implications for endodontic treatment. Accurate diagnosis through radiological examinations can help prevent complications during root canal therapy. A range of methods have been used to assess root canal morphology, including the evaluation of radiographs and clinical examination [13], root staining and clearing [14–16], cross‐sectional analysis of extracted teeth [17], resin injection techniques [18], radiographic methods enhanced with contrast media [19, 20], and micro‐computed tomography (micro‐CT) [21]. In clinical practice, the dentist may also employ magnification with loupes or an operating microscope. Conventional radiography (such as periapical radiographs) may not always provide sufficient detail to visualise unusual morphology. In such cases, cone‐beam CT (CBCT) offers distinct advantages because it enables precise three‐dimensional assessment of the tooth′s anatomical structure [22]. CBCT imaging is also valuable for evaluating morphological symmetry between the left and right sides in patients, which carries both scientific and clinical significance.

The aim of this clinical report is to describe the unique root anatomy of a mandibular second molar. We present a case involving a patient with bilateral second lower molars, each with four roots, whose complex morphology was assessed using CBCT. To the best of our knowledge, the bilateral occurrence of four‐rooted mandibular second molars has only been reported once before in the literature [23].

## 2. Case Presentation

**Figure 1:**
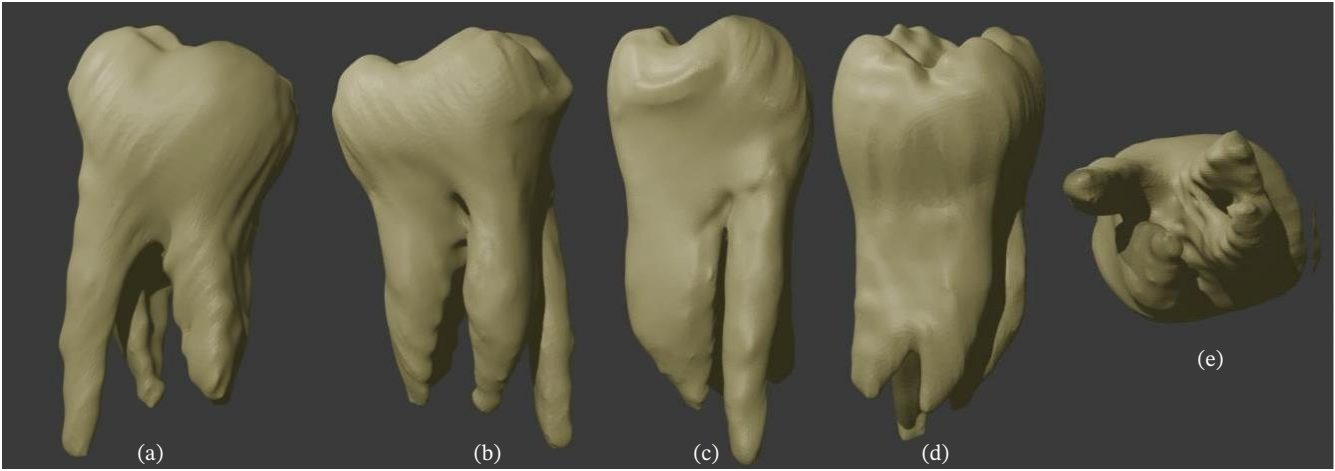
Three‐dimensional models of left second mandibular molar. (a) Buccal view, (b) lingual view, (c) mesial view, (d) distal view, and (e) apical view.

**Figure 2:**
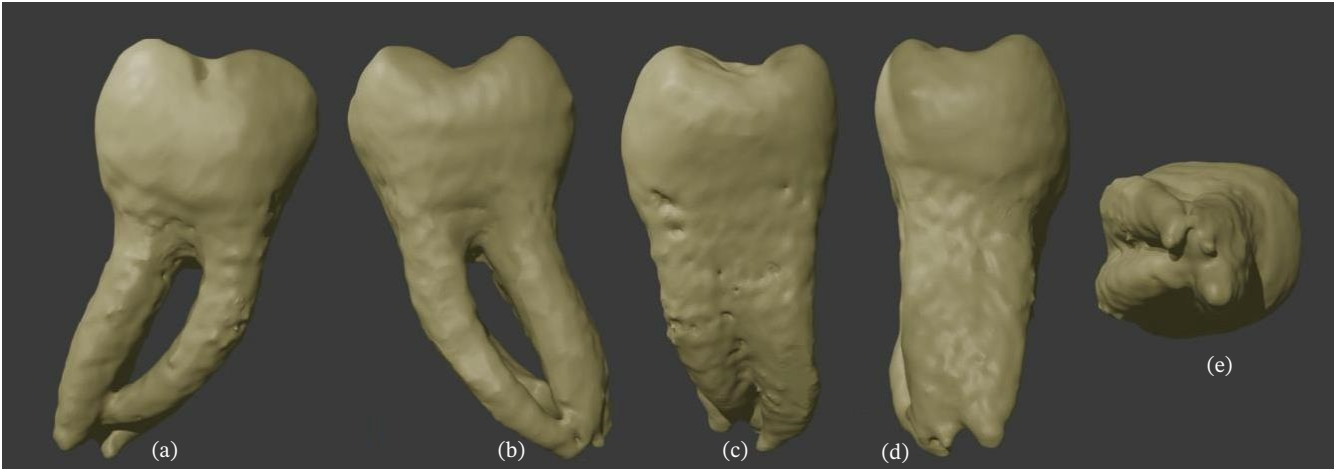
Three‐dimensional models of right second mandibular molar. (a) Buccal view, (b) lingual view, (c) mesial view, (d) distal view, and (e) apical view.

A 33‐year‐old Caucasian woman underwent CBCT examination of the mandibular teeth for diagnostic purposes. The patient provided informed consent, understanding the higher radiation dose compared with conventional radiographs. Exposure was minimized using a limited field of view and optimized scanning parameters, ensuring radiation safety. The examination was clinically justified for detailed assessment of tooth and root anatomy. During this examination, the endodontist noted the unusual anatomical structure of the lower second molars. Both the right and left mandibular second molars displayed a four‐root morphology, with the anomaly more pronounced on the left side. Intraoral clinical examination revealed crowns with a typical anatomical structure. The patient reported no complaints related to the second molars, and these teeth had never been treated. From the general medical history, the patient reported idiopathic scoliosis diagnosed at the age of 8, which had been surgically treated at 14. No other systemic diseases were reported. Assessment of the teeth′s morphological structure was performed in NewTom′s NNT software using CBCT scans acquired with the Cranex 3DX device (Soredex, Tuusula, Finland). In this case, the fields of view were 7.8 × 15 cm and 13 × 15 cm, with voxel sizes of 350 *μ*m and 400 *μ*m, respectively. To further illustrate the atypical morphology, three‐dimensional models of the mandibular second molars were generated in Exocad (Exocad GmbH, Darmstadt, Germany) (Figures 1 and 2).

Tooth length was measured from the root apex to the highest point of the corresponding cusp on the crown. The lower left second molar exhibited four roots—two mesial and two distal (Figure 3).

Figure 3Left second mandibular molar—axial views of root system. (a) Coronal part: visible mesiobuccal root (green arrow), mesiolingual root (yellow arrow), and distal root (white arrow). (b) Middle part: visible mesiobuccal root (green arrow), mesiolingual root (yellow arrow), and distal root (white arrow). (c) Apical part: visible mesiobuccal root (green arrow), mesiolingual root (yellow arrow), distobuccal root (blue arrow), and distolingual root (orange arrow).

**Figure:**
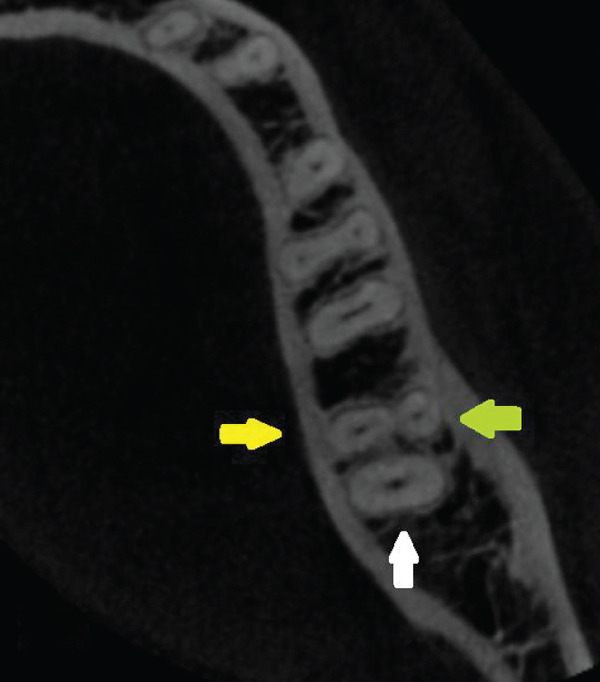
(a)

**Figure:**
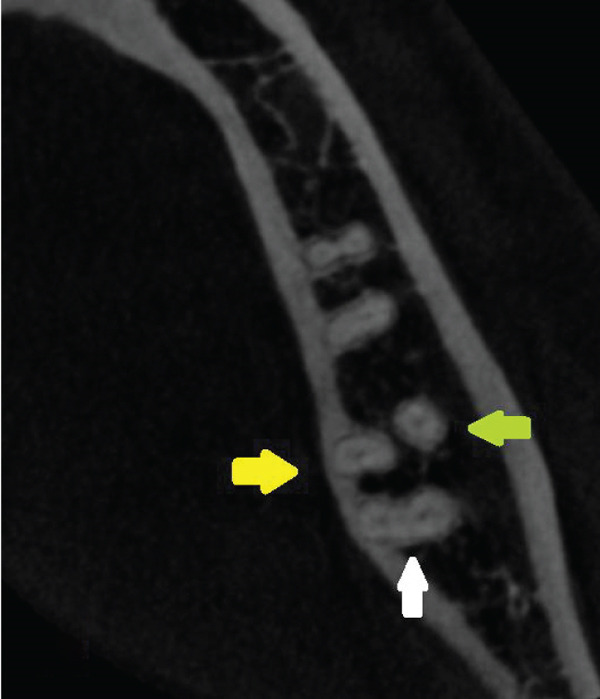
(b)

**Figure:**
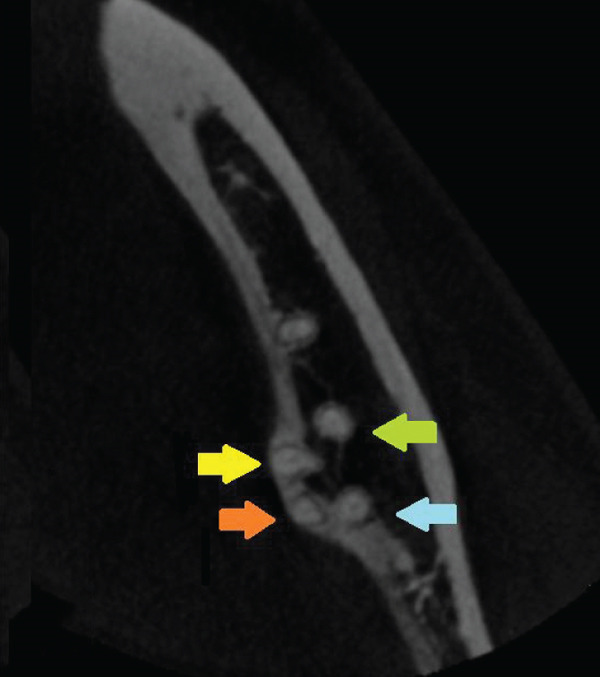
(c)

The root measurements were as follows. The mesiobuccal and mesiolingual roots measured 23.38 mm and 20.42 mm, respectively, with bifurcation occurring 7.79 mm from the apex. The distobuccal and distolingual roots measured 19.90 mm and 19.91 mm, respectively, with bifurcation occurring 4.23 mm from the apex. The lower right second molar showed a more fused structure; however, in the apical region, separation of the four roots was visible (Figure 4).

Figure 4Right second mandibular molar—axial views of root system. (a) Coronal part: visible mesial root (pink arrow) and distal root (white arrow). (b) Middle part: visible mesiobuccal root (green arrow), mesiolingual root (yellow arrow), and distal root (white arrow). (c) Apical part: mesiolingual root (yellow arrow), distobuccal root (blue arrow), and distolingual root (orange arrow).

**Figure:**
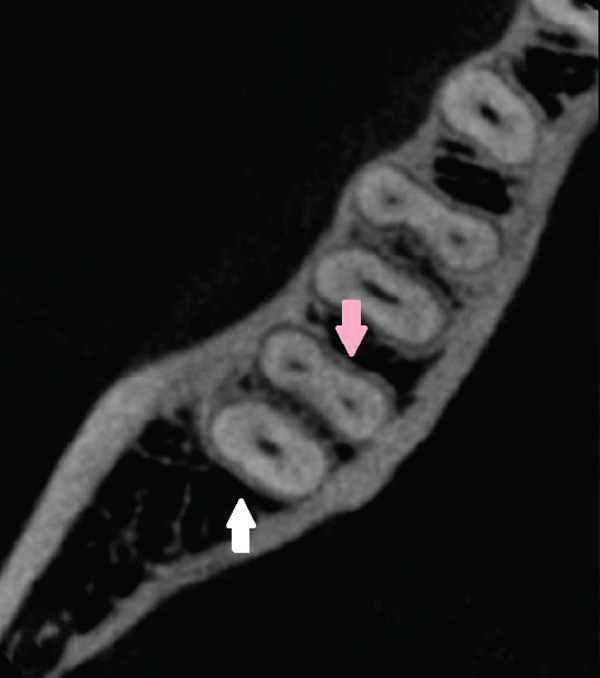
(a)

**Figure:**
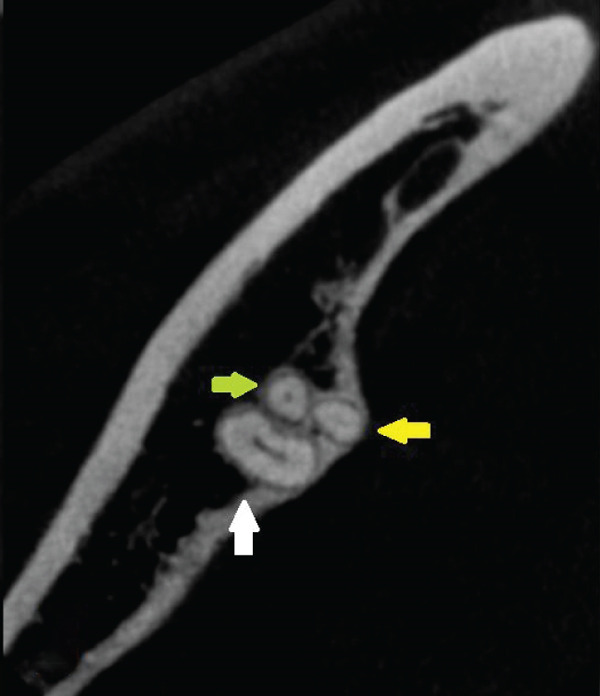
(b)

**Figure:**
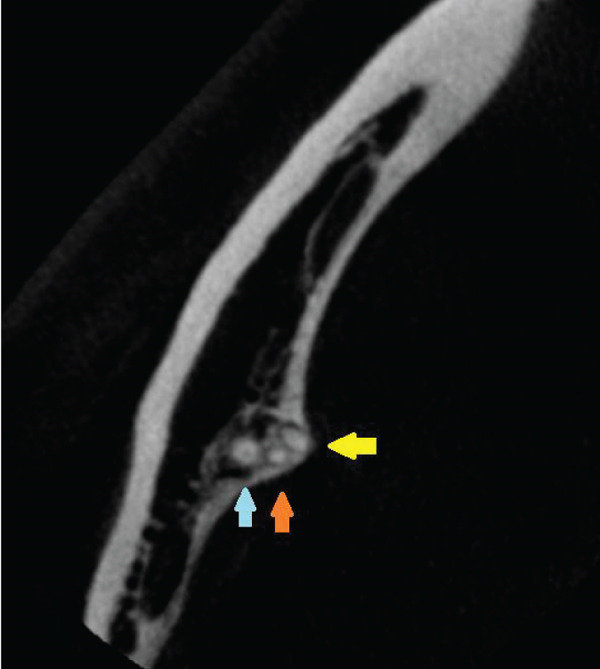
(c)

The mesiobuccal and mesiolingual roots measured 20.28 mm and 21.30 mm, respectively, with bifurcation occurring 5.84 mm from the apex. The distobuccal and distolingual roots measured 20.27 mm and 19.42 mm, respectively, with bifurcation occurring 2.20 mm from the apex.

## 3. Discussion

The development and morphology of tooth roots are largely determined by the proper functioning of Hertwig′s epithelial root sheath (HERS), which determines the direction of root growth, the number of roots, and the architecture of the furcation. The overall regulatory mechanisms governing tooth root formation remain largely unresolved. It is known, however, that reciprocal and sequential epithelial–mesenchymal interactions in early development lead to the formation of root dentin, cementum and periodontal tissues. HERS plays a crucial role in establishing the axis, number and architecture of the root system, and the entire process is coordinated by major signaling pathways—TGF‐*β*/BMP, Wnt, FGF, and SHH—which, together with numerous transcription factors, control the proliferation, differentiation and spatial organization of epithelial and mesenchymal cells during root odontogenesis. Disruptions in the integrity or segmentation of HERS, or disturbances in the activity of these regulatory pathways, may lead to root anomalies such as altered root length, abnormal branching, or variations in root number. Consequently, the genetic and molecular factors governing HERS function play a crucial role in determining individual differences in root system morphology [24–26].

The mandibular second molars most commonly have a two‐root structure, comprising a mesial and a distal root. Three‐rooted forms with an additional radix entomolaris or radix paramolaris are less frequently reported. Single‐rooted forms are usually associated with the presence of a C‐shaped canal, the prevalence of which varies according to the population studied [3, 4, 8]. In the literature, the four‐rooted form has been previously described only in a few cases. In studies by Erkman and Bektas on Iron Age human skeletons, the four‐rooted form of the mandibular second molar was found in 2% of the examined teeth [27]. Peiris et al. described the extraction of a single four‐rooted mandibular second molar in a Sri Lankan patient [11]. Bhardaj and Tarun also reported a case of a diagnosed and endodontically treated mandibular second molar with four roots in an Indian patient [28]. Martin et al. described the endodontic treatment of a four‐rooted left mandibular second molar in a 33‐year‐old Caucasian woman [10]. A 2‐year follow‐up of successful endodontic treatment of a four‐rooted mandibular second molar was reported by Altraroti et al. in a Saudi Arabian patient. In their report, the authors also reviewed studies of different populations involving a total of 2315 mandibular second molars, in which no four‐rooted forms were observed [29]. Endodontic retreatment of a four‐rooted mandibular second molar was reported by Irdis et al., who presented a case involving tooth 37 in a 45‐year‐old Indian patient. During the initial treatment, the presence of a fourth root and fourth root canal—the distobuccal root/canal—was not identified and therefore not treated [30]. Purra et al. also described a case of diagnosis and endodontic treatment of a four‐rooted mandibular second molar. Based on CBCT examination, they reported a variation differing from both previous cases and the present one: a mandibular second molar with three mesial roots and a single distal root, rather than two mesial and two distal roots [31]. An even rarer morphology of the mandibular second molar—the five‐rooted form—was described by Nistor et al. They reported a CBCT‐based diagnosis and endodontic treatment of tooth 47 in a 49‐year‐old Romanian patient. The tooth had five roots and five root canals: mesiolingual, middle mesial, mesiobuccal, distobuccal, and distolingual [12]. Bilateral occurrence of four‐rooted mandibular second molars has only been reported by Shinde et al. Their case report described a 30‐year‐old patient with bilateral four‐rooted mandibular first and second molars, as well as bilateral three‐rooted mandibular first and second premolars [23].

Table 1 summarises the reported cases of a four‐rooted mandibular second molar available in the literature [10, 12, 14, 27–31].

**Table 1:** Reported cases of four‐rooted mandibular second molar.

Authors (year)	Research method	Population(country)	Sex	Age (years)	Number of teeth	Position of roots	Number of root canals
Peiris et al. (2009) [11]	Clinical diagnosis	Sri Lanka	Female	23	1	MB, ML, DB, DL	4
Erkman and Bektas (2012) [27]	Clinical diagnosis	Dilkaya, Eastern Anatolia(Iron Age)	N/A	12–13	1	MB, ML, DB, DL	N/A
Purra et al. (2013) [31]	Clinical and radiographic diagnosis (CBCT)	India	Male	21	1	MB, MM, ML, DT	4
Martins et al. (2014) [10]	Clinical and radiographic diagnosis (CBCT)	Portugal	Female	33	1	MB, ML, DB, DL	4
Irdis et al. (2014) [30]	Clinical and radiographic diagnosis (CBCT)	India	Male	45	1	MB, ML, DB, DL	4
Shinde et al. (2016) [23]	Clinical and radiographic diagnosis (CBCT)	India	Male	30	2(bilateral)	MB, ML, DB, DL	4
Bhardaj and Tarun (2017) [28]	Clinical and radiographic diagnosis (periapical radiograph)	India	Male	60	1	MB, ML, DB, DL	5
Altraroti et al. (2021) [29]	Clinical and radiographic diagnosis (CBCT)	Saudi Arabia	Female	23	1	MB, ML, DB, DL	4
Nistor et al. (2023) [12]	Clinical and radiographic diagnosis (CBCT)	Romania	Female	49	1(five roots)	MB, MM, ML, DB, DL	5

Abbreviations: CBCT, cone‐beam computed tomography; DB, distobuccal; DL, distolingual; DT, distal N/A, not available; MB, mesiobuccal; ML, mesiolingual; MM, middle mesial.

In larger population studies, four‐rooted second molars were found to constitute only a small percentage. Table 2 presents studies evaluating the morphology of mandibular second molars in different populations [14, 31–56]. Buchanan et al., in a study of 386 teeth from a South Black African population, reported two four‐rooted forms (0.5%) [56]. Shemesh et al., investigating the occurrence of three‐rooted and four‐rooted mandibular second molars, identified eight four‐rooted cases, representing 0.55% of 2694 teeth examined in an Israeli population [35]. Demibruga et al. described eight four‐rooted mandibular second molars in a Turkish population, accounting for 0.86% of 925 teeth [34]. Celikten et al., also studying a Turkish population, reported one case of a four‐rooted mandibular second molar, which constituted 0.2% of the 421 teeth examined [37]. In 2017, von Zuben et al. assessed the worldwide prevalence of C‐shaped mandibular second molars and found one tooth with four roots in an English population (0.3% of 400 teeth) [42]. In a Chinese population, Yang et al. described three four‐rooted mandibular second molars, representing 0.25% of 1200 teeth examined [51]. Khawaja et al., in a study of an Emirati population, reported four‐rooted forms in 0.6% of 508 examined teeth [50].

**Table 2:** Analysis of the morphology of the lower second molars in different populations.

Author (year)	Population (country)	Method	Number of roots						C‐shaped root
			One	Two	Three	Four	Fused	Total	
Peiris et al. (2007) [14]	Sri Lanka	Clinical examination	4 (4.0)	96 (96.0)	—	—	—	100 (100)	4 (4.0)
Al‐Qudah et al. (2009) [32]	Jordan	Clinical examination	45 (12.7)	291 (82.0)	—	—	19 (5.4)	355 (100)	37 (10.4)
Zhang et al. (2011) [33]	China	CBCT	35 (22.0)	119 (76.0)	3 (2.0)	—	—	157 (100)	10 (29.0)
Demirbuga et al. (2013) [34]	Turkey	CBCT	12 (1.29)	790 (85.4)	32 (3.45)	8 (0.86)	83 (8.96)	925 (100)	38 (4.1)
Shemesh et al. (2015) [35]	Israel	CBCT	N/A	26 (1.78)	N/A	8 (0.55)	N/A	1464 (100)	N/A
Felsypremila et al. (2015) [36]	India	CBCT	28 (8.5)	286 (88.8)	8 (2.5)	—	—	322 (100)	26 (8.1)
Celikten et al. (2016) [37]	Turkey	CBCT	7 (1.6)	403 (95.7)	2 (0.4)	1 (0.2)	—	421 (100)	8 (1.9)
Kim et al. (2016) [38]	Korea	CBCT	790 (41.15)	1102 (57.4)	14 (0.72)	—	—	1920 (100)	770 (40.0)
Madani et al. (2017) [39]	Iran	CBCT	26 (17.6)	120 (81.6)	1 (0.6)	—	—	147 (100)	26 (17.6)
Pérez‐Heredia et al. (2017) [40]	Spain	CBCT	20 (16.5)	101 (83.5)	—	—	—	121 (100)	—
Martins et al. (2017) [41]	Portugal	CBCT	95 (14.2)	554 (83.1)	18 (2.7)	—	—	667 (100)	—
von Zuben et al. (2017) [42]	Brazil	CBCT	33 (8.3)	364 (91.0)	3 (0.8)	—	—	400 (100)	27 (6.8)
	China	CBCT	188 (46.5)	211 (52.8)	3 (0.8)	—	—	400 (100)	176 (44.0)
	England	CBCT	38 (9.5)	348 (87.0)	13 (3.3)	1 (0.3)	—	400 (100)	31 (7.8)
	India	CBCT	51 (12.8)	348 (87.0)	1 (0.3)	—	—	400 (100)	49 (12.3)
	Mexico	CBCT	65 (16.3)	330 (82.5)	5 (1.3)	—	—	400 (100)	57 (14.2)
	Portugal	CBCT	45 (11.3)	344 (86.0)	11 (2.8)	—	—	400 (100)	33 (8.3)
	South Africa	CBCT	39 (9.8)	353 (88.3)	8 (2.0)	—	—	400 (100)	37 (9.3)
	Spain	CBCT	46 (11.5)	349 (87.3)	5 (1.3)	—	—	400 (100)	44 (11.0)
	United States	CBCT	48 (12.0)	341 (85.3)	11 (2.8)	—	—	400 (100)	45 (11.3)
Pawar et al. (2017) [43]	India	CBCT	—	780 (79.35)	74 (7.53)	—	129 (13.12)	983 (100)	129 (13.12)
Martins et al. (2018) [44]	Portugal	CBCT	98 (14.3)	571 (83.1)	18 (2.6)	—	—	687 (100)	—
Donyavi et al. (2019) [45]	Iran	CBCT	41 (9.2)	397 (88.8)	9 (2.0)	—		447 (100)	41 (9.2)
Pan et al. (2019) [46]	Malaysia	CBCT	183 (48.7)	192 (51.1)	1 (0.27)	—	—	376 (100)	183 (48.7)
Abarca et al. (2020) [47]	Chile	CBCT	70 (13.7)	442 (86.3)	—	—	—	512 (100)	56 (10.9)
Gomez et al. (2021) [48]	Venezuela	CBCT	23 (12.1)	162 (85.3)	5 (2.6)	—	—	190 (100)	37 (19.5)
Senan et al. (2021) [49]	Yemen	CBCT	4 (0.8)	448 (89.6)	3 (0.6)	—	45 (9.0)	500 (100)	45 (9.0)
Khawaja et al. (2021) [50]	United Arab Emirates	CBCT	N/A (2.4)	N/A (78.3)	N/A (0.8)	N/A (0.6)	—	508 (100)	N/A (17.9)
Yang et al. (2022) [51]	China	CBCT	270 (44.9)	328 (54.6)	2 (0.16)	3 (0.25)	—	1200 (100)	430 (35.8)
Almansour et al. (2022) [52]	Saudi Arabia	CBCT	—	286 (94.1)	5 (1.6)	—	13 (4.3)	304 (100)	13 (4.3)
Mantovani et al. (2022) [53]	Brazil	CBCT	96 (8.00)	1082 (90.17)	22 (1.83)	—	—	1200 (100)	59 (4.91)
Saber et al. (2023) [54]	Egypt	CBCT	57 (16.3)	292 (83.4)	1 (0.3)	—	—	350 (100)	45 (12.9)
Guo et al. (2023) [55]	China	CBCT	208 (36.62)	358 (63.03)	2 (0.35)	—	—	568 (100)	202 (35.56)
Buchanan et al. (2023) [56]	South Africa	CBCT	24 (6.2)	354 (91.7)	6 (1.6)	2 (0.5)	—	386 (100)	22 (5.7)

*Note:* Data are presented as n (%).

Abbreviations: CBCT, cone‐beam computed tomography and N/A, not available.

The analysis of the literature shows that in clinical practice, dentists may encounter considerable morphological diversity in teeth requiring endodontic treatment. Accurate knowledge of tooth anatomy is one of the fundamental conditions for successful therapy. With the development of modern radiological diagnostics (CBCT), it is now possible to detect structural irregularities at the planning stage of endodontic treatment. CBCT enables a three‐dimensional evaluation of root and canal anatomy, facilitating the precise planning of endodontic access and the identification of all canal orifices. In cases of developmental anomalies, such as a four‐rooted mandibular second molar, it is essential to appropriately modify the conventional access approach to locate all canals and ensure straight‐line access for endodontic instrumentation. Moreover, the use of CBCT in access planning allows for accurate determination of the direction, depth, and width of the access cavity, minimizing the risk of missed canals. Such individualized access modification, based on a thorough understanding of anatomical variations and confirmed by imaging, is a critical determinant for the success of endodontic treatment in teeth with additional roots and additional roots canals. In our case, teeth 37 and 47 did not require treatment, and their atypical four‐root structure was identified during a routine CBCT control scan. By contrast, in most cases reported in the literature, the diagnosis of a four‐rooted mandibular second molar was made during primary endodontic treatment or retreatment. Although CBCT is not yet a routine part of endodontic diagnostics, in difficult or unusual cases it should be considered an essential tool for accurate diagnosis.

## Author Contributions

**Agnieszka Chamarczuk** and **Mariusz Lipski** wrote the main manuscript text. **Agnieszka Chamarczuk** and **Laurentia Schuster** prepared the tables and figures. **Mariusz Lipski** and **Till Dammaschke** performed content verification and revisions.

## Funding

This work was supported by Pomorski Uniwersytet Medyczny w Szczecinie, 10.13039/501100008781.

## Disclosure

All authors reviewed the manuscript.

## Consent

Written informed consent for the publication of the case details and related clinical images was voluntarily obtained from the patient. A copy of the written consent is available for review by the editor‐in‐chief of this journal.

## Conflicts of Interest

The authors declare no conflicts of interest.

## Data Availability

Data sharing not applicable to this article as no datasets were generated or analysed during the current study.
